# Learning from prostate cancer statistics

**DOI:** 10.3322/caac.70037

**Published:** 2025-09-29

**Authors:** Ruth Etzioni, Lukas Owens

**Affiliations:** ^1^ Fred Hutchinson Cancer Center Seattle Washington USA

The population represents the ultimate uncontrolled experiment, yet data on cancer statistics provide an opportunity to learn about real‐world outcomes of cancer control activities and policies. In the case of prostate cancer, population data have been critically important in generating and confirming hypotheses about the impacts of screening and treatment advances on the population burden of the disease. Tracking prostate cancer statistics—incidence, mortality, and survival—and how they change over time is thus a prerequisite for understanding the success (or lack thereof) of efforts to control this most common cancer in American men. But population statistics are multifactorial; explaining them requires also considering their many potential drivers and the mechanisms by which disease control efforts play out in the population.

Consider the example of prostate cancer incidence, prominently reported in this issue’s update on prostate cancer statistics.^1^ Prostate cancer incidence is influenced by prostate‐specific antigen (PSA) screening rates in the population. Incidence increased dramatically during the early years of the PSA screening era, prompting concerns that screening was leading to overdiagnosis. Although overdiagnosis did indeed turn out to be a problematic outcome of screening, work by Feuer and Wun^2^ in the early 1990s assured that increases in disease incidence were to be expected when a new screening test was adopted at the population level. The mechanism—initial depletion of the prevalent pool of cases by the screening test—leads to a predicted peak in incidence followed by declines because of the absence in the prevalent pool of those previously detected cases. Feuer and Wun demonstrated that the height and duration of the peak would be driven by the lead time, which is the time by which screening advances disease diagnosis. The lead time is critical not only in the timing of incidence swings after the adoption of screening but also in the delay until any effects of screening on disease mortality are observed. And the average lead time associated with prostate cancer screening is not short—estimates based on the first decade of PSA screening place the mean lead time between 5 and 7 years.^3^

The update of prostate cancer statistics in this issue of *CA: A Cancer Journal for Clinicians* highlights more recent incidence trends, specifically the persistence of recent increases overall and in advanced‐stage disease. These trends have generated concern because they are what one would expect in a population abandoning screening. Indeed, studies tracking both incidence and screening patterns have been on the alert for such trends, particularly after the issuance of the D recommendation against routine prostate cancer screening for all ages by the US Preventive Services Task Force in 2012.^4^ Although some modest reductions in prostate cancer screening were detected after this recommendation,^5^ no studies have linked these patterns with the recent incidence trends. Definitively doing so is very challenging, but a necessary condition is that the pattern of incidence comports with what would be expected given the mechanistic effects of screening in the population and given other potentially influential factors.

From a mechanistic perspective, a reduction in screening would be expected to lead to a reversal of the patterns observed at the beginning of the screening era, but the timing and magnitude of the reversal would depend on the extent of the reduction as well as on the lead time. A mechanistic model projecting incidence under wholesale cessation of PSA screening suggested that incidence would initially drop considerably but then begin to increase soon thereafter.^6^ Late‐stage incidence would not be expected to drop but rather would be expected to increase. After the 2012 US Preventive Services Task Force recommendation, incidence did drop, but this drop simply accelerated a decline that was already underway. Distant‐stage incidence has since trended upward. Thus, from a mechanistic perspective, the recent late‐stage and overall incidence trends are consistent with reduced PSA testing. However, other potential contributors to these trends merit consideration, including the roles of changes in the way screening is conducted and the methods by which prostate cancer is staged.

Since the adoption of PSA screening in the population, there have been many changes in the ways men are screened and diagnosed. A key factor driving incidence under screening has been biopsy technique, which has evolved significantly over time. Increases in biopsy cores from four or six to 10 or 12 were followed by efforts to reduce diagnosis of clinically insignificant cancers, primarily through reflex testing (e.g., using magnetic resonance imaging). The role of these changes in the diagnosis of disease and in the profile of detected cancers merits consideration in explaining trends in disease incidence. In addition, use of more advanced imaging and improvements in pathology technology may upstage cases and produce apparent increases in late‐stage diagnosis.

Recent analyses examine the competing explanations for the observed incidence trends. Owens et al.^7^ studied recent trends in age and PSA at initial diagnosis of prostate cancer and concluded that changes in these quantities were more consistent with a delay in detection (for example, because of cessation of screening) than with upstaging at the time of diagnosis. Nyame et al.^8^ used a mechanistic model to project the impact of decreased screening utilization on advanced‐stage incidence and also concluded that the data were consistent with an effect of reductions in screening. These studies lend additional support to the assessment that current trends in prostate cancer incidence and late‐stage incidence are likely caused by a drop in population screening.

This discussion of interpreting trends in prostate cancer incidence showcases the multifactorial nature of population cancer statistics. Changes in diagnostic technologies and practices will affect observed trends in incidence and survival, but some of the changes may be artifactual. Understanding the mechanistic implications of changes in screening practices is necessary to properly interpret prostate cancer trends. Increases in screening will generally lead to contemporaneous incidence increases but later declines. Reduced screening in younger age groups will have implications for late‐stage incidence in older age groups. And uptake of novel technologies for disease staging, such as prostate‐specific membrane antigen–positron emission tomography/computed tomography, will inevitably change the incidence and indeed the very definition of advanced‐stage disease.^9^ Recognizing how mechanistic drivers produce disease patterns over time and expanding consideration of explanations beyond simple, proximal factors will be necessary to avoid oversimplifying or jumping to foregone conclusions when learning from prostate cancer statistics.

## CONFLICT OF INTEREST STATEMENT

Ruth Etzioni owns stock in Seno Medical outside the submitted work. Both authors report grants/contracts from the National Cancer Institute.
